# A large calibrated database of hand movements and grasps kinematics

**DOI:** 10.1038/s41597-019-0349-2

**Published:** 2020-01-09

**Authors:** Néstor J. Jarque-Bou, Manfredo Atzori, Henning Müller

**Affiliations:** 1 Department of Mechanical Engineering and Construction, Universitat Jaume I, Castellon de la Plana, Spain; 2 Information Systems Institute, University of Applied Sciences Western Switzerland (HES-SO Valais), Sierre, Switzerland; 3 Medical faculty, University of Geneva, Geneva, Switzerland

**Keywords:** Bone, Skeleton, Biomedical engineering

## Abstract

Modelling hand kinematics is a challenging problem, crucial for several domains including robotics, 3D modelling, rehabilitation medicine and neuroscience. Currently available datasets are few and limited in the number of subjects and movements. The objective of this work is to advance the modelling of hand kinematics by releasing and validating a large publicly available kinematic dataset of hand movements and grasp kinematics. The dataset is based on the harmonization and calibration of the kinematics data of three multimodal datasets previously released (Ninapro DB1, DB2 and DB5, that include electromyography, inertial and dynamic data). The novelty of the dataset is related to the high number of subjects (77) and movements (40 movements, each repeated several times) for which we release for the first time calibrated kinematic data, resulting in the largest available kinematic dataset. Differently from the previous datasets, the data are also calibrated to avoid sensor nonlinearities. The validation confirms that the data are not affected by experimental procedures and that they are similar to data acquired in real-life conditions.

## Abstract (summary)

**Table:** 

Measurement(s)	movement quality • grasps kinematics • muscle electrophysiology trait
Technology Type(s)	sensor • electromyography
Factor Type(s)	type of movement • joint movement repetition • age • sex • left-handed or right-handed • weight • height • body mass index
Sample Characteristic - Organism	Homo sapiens

Machine-accessible metadata file describing the reported data: 10.6084/m9.figshare.11341679

## Background & Summary

The hand is a complex functional limb including over 30 muscles and more than 20 joints that allow performing a wide range of activities with a high level of precision. Kinematics is essential for hand functioning. The human hand has a linear actuator structure and it has control requirements that differ from the most common designs used to replicate it^1,2^. The analysis of complex hand movements is useful for several applications, including robotics^3,4^ (to improve grasping by manipulators), 3D modelling^5^ (to develop more realistic models of the hand for movies or computer games), rehabilitation and physiotherapy^6–8^ (to improve hand rehabilitation), bioengineering, medicine and neuroscience (to better understand human hand movements, also in relationship to muscular and kinematic synergies^9,10^).

Although studies are improving the understanding of hand kinematics^11–15^, scientific research in this field is still often affected by several limitations. First, most of the studies involve a small number of subjects (up to 10 subjects to our knowledge^13^), lacking the possibility to generalize the results. Second, the studies often involve a small number of grasps, (up to 25 grasps to the best of our knowledge^13^), lacking result completeness. Third, usually only postural movements were considered, without taking into account the entire movement, while “reach to grasp” and release are important phases in grasps modelling. Finally, most studies are based on raw instrumented glove data, which do not provide the linear outputs required to obtain reliable joint angles and can invalidate kinematic models obtained without a specific and accurate calibration method^16,17^.

Hand movements can be measured with different methods, but most of them fail when capturing kinematics while performing ADL (Activities of Daily Living). Goniometers do not allow for the simultaneous measurement of all DoFs (Degrees of Freedom). Electromagnetic systems are susceptible to magnetic and electrical interference from metallic objects in the environment. Marker-based optical systems can be used only within the area covered by the cameras, require a substantial amount of time to setup the markers, and markers often become occluded during the recording of tasks. Recently, portable and relatively low-cost devices became available, such as the Leap Motion controller system. However, these systems lack of accuracy to obtain reliable kinematic data during the performance of ADL^18^. At this point, instrumented gloves seem to be the most effective method for collecting data without occlusion problems and are among the most frequently used methods to collect data from finger joints and hand movements. However, the use of data gloves is not always straightforward. First, the response of the sensors can change depending on the size of the hand of the user. Second, the sensors can have non-linear relationships with the joint anatomical angles^16^, due to their position or due to the influence of other joint movements. Therefore, calibration processes are fundamental to obtain reliable gains for the sensors that record each degree of freedom.

Subject-specific data glove calibration procedures are time consuming. Thus, it is not easy to include them in data acquisition protocols (that are often already long and tiring). This consideration is true for healthy controls and particularly for patients and persons affected by disabilities, for which data acquisitions can be even more stressful and physically demanding.

A recently presented calibration method assures the possibility to calibrate the kinematic data recorded with a data glove in post-processing^17^. The method was described having a reasonable maximum precision error (below 5 degrees), thus it can improve the accuracy with which hand kinematics and anatomical angles are quantified.

In this work we apply the post-processing calibration method to kinematic data from 77 intact subjects included in the Ninapro (Non Invasive Adaptive Prosthetics) database (Ninapro Repository (http://ninapro.hevs.ch) and Zenodo^19^ (10.5281/zenodo.3354437)). The novelty of the paper is related to the high number of subjects and movements and to the fact that the data are for the first time calibrated. The 77 subjects performed 40 hand movements and grasps plus rest, leading to our knowledge to the biggest hand kinematics dataset currently available. To obtain the hand anatomical angles, an across-subject calibration procedure^17^ was applied.

This dataset aims at allowing worldwide research groups to study hand kinematics. The calibrated data are expected to foster the progress in many scientific domains, such as medicine, neuroscience, rehabilitation, physiotherapy, robotics, prosthetics and computer aided model design, leading for instance to a better understanding of human hand movements, improved rehabilitation protocols, robotic grasps that better correspond to human’s and more realistic 3D graphical models.

In conclusion, the kinematic dataset Ninapro DB9 improves the scientific state of the art with the most comprehensive reference for kinematic data existing to the best of our knowledge. The technical validation section verifies that the data are similar to data acquired in real-life conditions by statistical analyses and the visual inspection of the 3D hand model representations.

## Methods

### Subjects and ethical requirements

The data were recorded from 77 intact subjects (56 males, 21 females; 69 right handed, 8 left handed; age 28.80 ± 3.96 years, see Table 1) and were originally released as part of three multimodal datasets (Ninapro DB1, DB2, DB5) including uncalibrated kinematic data^20,21^ of respectively 27, 40 and 10 subjects.

**Table 1:** Information on the input data used for the data set created in this paper.

	Kinematic Ninapro
Available subjects	77
Considered subjects	77
Males	56
Females	21
Right-handed	69
Left-handed	8
Avg. Age (years)	28.8 ± 3.96
Avg. Height (cm)	172.6 ± 9.48
Avg. Weight (kg)	69.5 ± 11.97
Avg. BMI (Kg/m^2^)	23.06 ± 3.18

Before the data acquisition began, each subject was given a thorough written and oral explanation of the experiment, including the associated risks; the subject was then asked to sign an informed consent form. The experiment was conducted according to the principles expressed in the Declaration of Helsinki (http://www.wma.net/en/20activities/10ethics/10helsinki) and it was approved by the Ethics Commission of the Canton of Valais (Switzerland).

### Acquisition setup

**Fig. 1:**
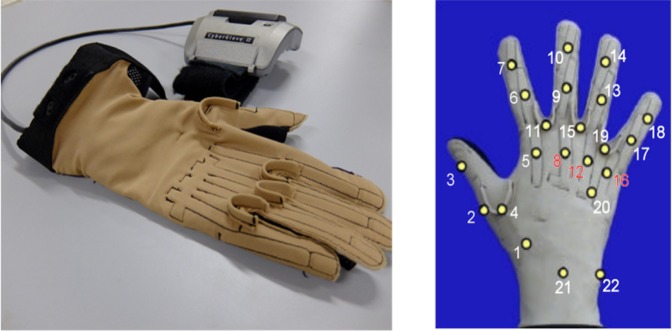
Cyberglove II device and the placement of the 22 sensors.

Hand kinematics was measured for all subjects using a 22-sensor CyberGlove II data glove (CyberGlove Systems LLC, www.cyberglovesystems.com). The CyberGlove is a motion capture data glove, instrumented with joint-angle measurement sensors. It uses proprietary resistive bend-sensing technology to transform hand and finger motions into real-time digital joint-angle data. Data from the CyberGlove were transmitted over a Bluetooth-tunnelled serial port at slightly less than 25 Hz. Each data sample provided was associated with an accurate timestamp using Windows performance counters. Then, the data streams were super-sampled to the highest sampling frequency in the acquisition setup (100 Hz, 2 kHz and 200 Hz respectively for Ninapro DB1, DB2 and DB5). The number and the corresponding position of each Cyberglove sensor is shown in Fig. 1.

Electromyography data are also available for each subject in the original multimodal datasets (Ninapro DB1, DB2 and DB5)^20,21^. We invite the users willing to use calibrated kinematic, sEMG or other modalities together to read these papers for a detailed description of the acquisition setups (including sEMG, inertial, and force exertion data), the sEMG sensors (respectively including 10 OttoBock MyoBock 13E200, 12 Delsys Trigno Wireless electrodes and 2 Thalmic Myo armbands (http://www.thalmic.com/) and the synchronization procedures^20,21^.

### Acquisition protocol

Subjects were asked to sit at a desk in an office chair, adjusted to match the maximum comfort, while comfortably resting their arms on the desktop. A laptop in front of the subject provided visual stimuli for each task, while also recording data from the data acquisition devices. The experiment is divided into one training part and several exercises addressing different types of movements, interrupted by rest time in order to avoid muscular fatigue.

**Fig. 2:**
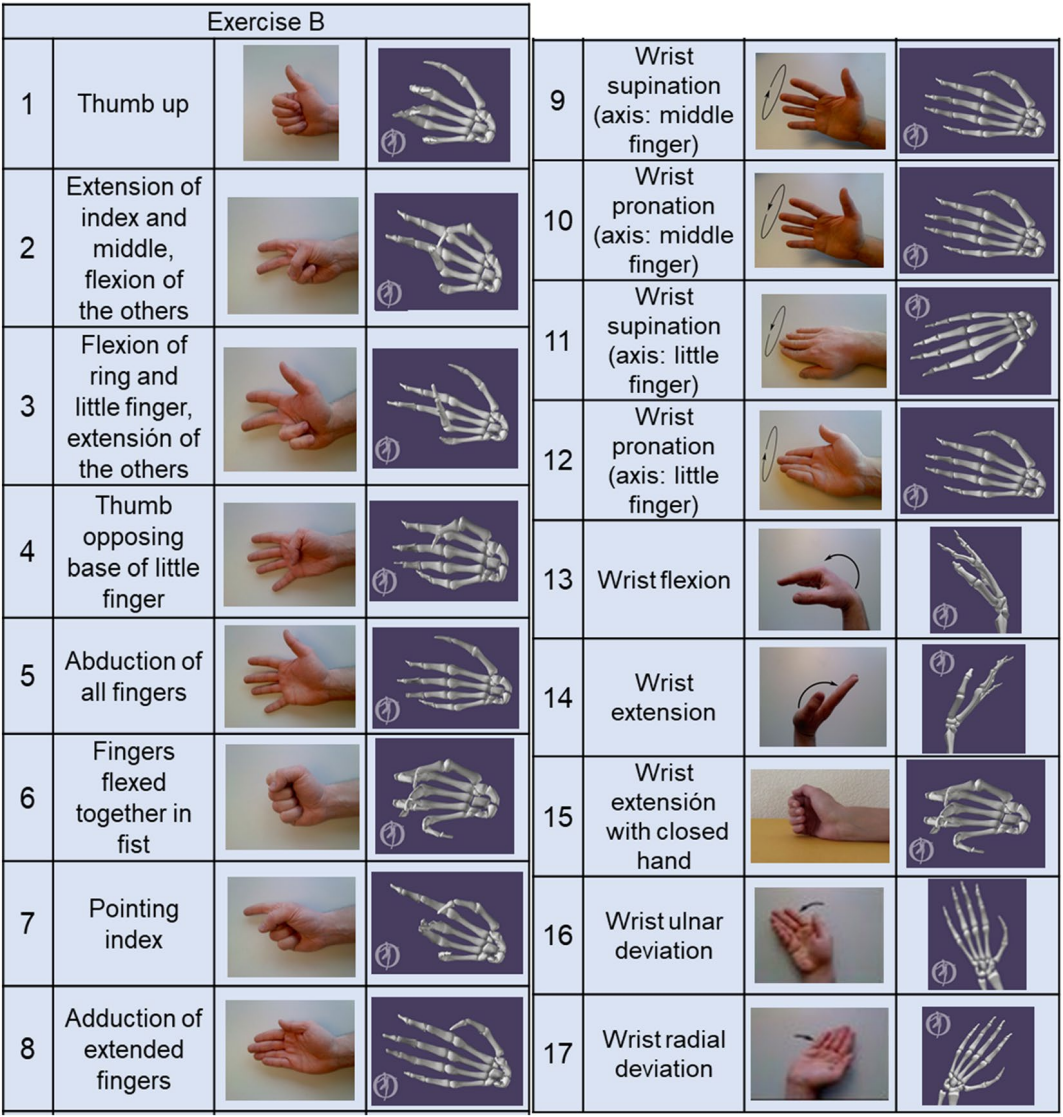
Graphical representation of each movement for the Exercises B: 8 isometric and isotonic hand configurations and 9 basic movements of the wrist.

**Fig. 3:**
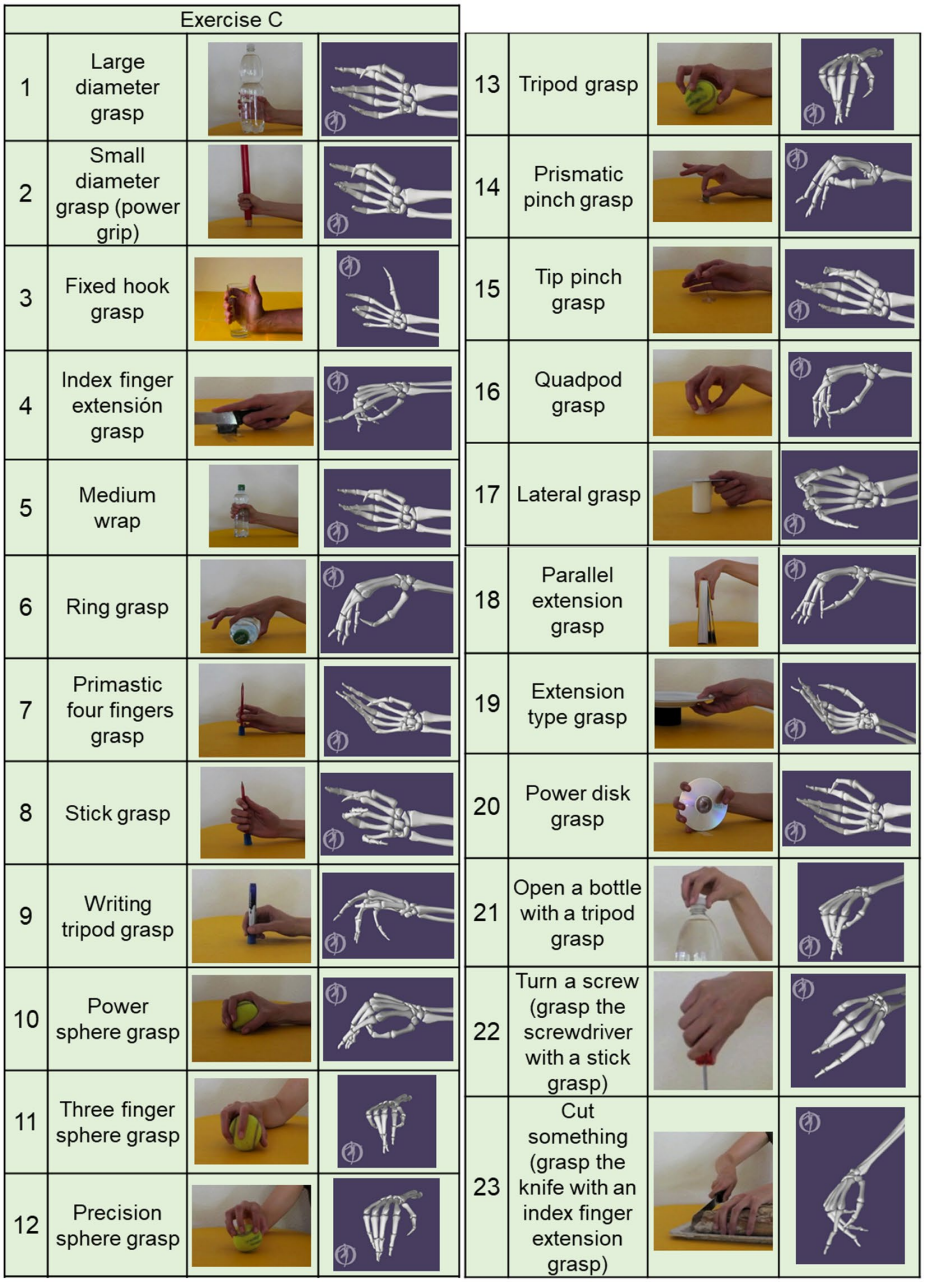
Graphical representation of each movement for the Exercises C: 23 grasping and functional movements (everyday objects are presented to the subject for grasping, in order to mimic daily-life actions).

The exercises included in the kinematic dataset consist of 40 hand movements plus rest and correspond to exercise B and C described by Atzori *et al*.^20^. Exercise B (Fig. 2) consists of 8 isometric and isotonic hand configurations and 9 basic movements of the wrist; Exercise C (Fig. 3) consists of 23 grasping and functional movements (everyday objects are presented to the subject for grasping, in order to mimic daily-life actions). The Ninapro DB1 and DB5 datasets originally included exercises A, B and C^20^, while DB2 included exercises B, C and D^21^.

During the exercises performed using the Cyberglove II, the subjects were asked to mimic the actions shown in short videos on the screen of the laptop with their right hand. All the subjects were asked to concentrate on mimicking the movements rather than on exerting high forces. The set of movements was selected from the hand taxonomy, robotics and rehabilitation literature with the aim of corresponding to the hand movements encountered in activities of daily living (ADL)^22–25^. Each movement repetition lasted 5 seconds, and it was followed by 3 seconds of rest. The sequence of movements was not randomized in order to encourage repetitive, almost unconscious movements.

The calibrated kinematic dataset is available online on the official Ninapro repository as Ninapro DB9 (http://ninapro.hevs.ch/db9) and on Zenodo^19^.

### Signal processing

Several signal processing steps were performed before making data publicly available in the repositories. These steps include relabelling and joint angle computation. The raw glove data are also available on Zenodo^19^.

### Relabelling

The movements performed by the subjects may not perfectly match with the stimuli proposed by our software due to human reaction times and experimental conditions. The resulting erroneous movement labels were corrected by applying movement detection algorithms offline, such as the generalized likelihood ratio algorithm^26^ and the Lidierth threshold based algorithm^27,28^.

### Joint angles

Calibration is required to keep into account some issues related to datagloves, such as non-linearities. Therefore, joint angles were computed by transforming the raw data according to specific gain values computed on the basis of a post-processing calibration protocol^17^ that was recorded for 10 subjects. The protocol consists of recording 65 poses and guided movements to obtain the sensor gains and some corrections to cross-coupling effects for specific anatomical angles. Thirteen calibration trials correspond to the calibration of wrist flexion/extension (WRIST_F) and deviation (WRIST_A). The trials include postures corresponding to different, mixed wrist flexion and deviation levels of the wrist. The sensor gains for the wrist are shown in Table 2.

**Table 2:** Information on the sensor gains used for the calibration of the wrist gauges. Sx corresponds to the sensor number, according to Fig. 1.

	Sensor gains			Anatomical angle calculation
	G1	G2	G3	
WRIST_F	0.008	−190.451	—	S21^2^*G1 + G2
WRIST_D	−1.020	0.467	75.336	S22*G1 + S21*G2 + G3

Twenty-eight calibration trials correspond to the calibration of 14 flexion sensors (two static postures per sensor) that measure the flexion of the metacarpophalangeal joints (MCP_F), proximal interphalangeal joints (PIP_F) and distal interphalangeal joints (DIP_F) (respectively 1 to 5 corresponding from thumb to little digit). The gains of these flexion sensors assume a linear relationship between the flexion angle at these joints and the glove output signals. The sensor gains for the flexor/extensor digit gauges are shown in Table 3.

**Table 3:** Information on the sensor gains used for the calibration of the flexion/extension gauges.

	Sensor gains					Anatomical angles calculation
	Digit1	Digit2	Digit3	Digit4	Digit5	
DIP_F	—	1.08	1.82	2.08	1.26	*Sensor gain·sensor gauge value
IP/ PIP_F	1.19	0.91	1.07	1.57	0.89	
MCP_F	0.562	0.91	0.95	0.97	1.06	

Eighteen calibration trials correspond to the calibration of 3 abduction sensors, corresponding to relative abduction of MCP of the fingers (MCP2-3, MCP3-4, MCP4-5). Due to the placement of the sensors on the glove, the output signal of the abduction sensors varies when the adjacent MCP joints are also flexed. This happens also when there is no variation of the abduction angles, requiring to correct the abduction output angles^16^. In order to correct this effect on the abduction angles, the gains of these sensors assume a second order polynomial relationship between the flexion angles of adjacent MCP joints and the glove output signals. The sensor gains for the abduction/adduction digit gauges are shown in Table 4.

**Table 4:** Information on the sensor gains used for the calibration of the abduction/adduction gauges. Sx corresponds to the sensor number, according to Fig. 1.

	Sensor gains						Anatomical angle calculation
	G1	G2	G3	G4	G5	G6	
MCP2-3_A	−0.503	−0.071	−0.004	0.091	−0.010	0.014	*G*1·*S*11 + (G2·*S*5 + G3·*S*8 + G4·*S*5^2^ + G5·*S*8^2^ + G6·*S*5·*S*8)
MCP3-4_A	0.229	−0.169	0.006	0.167	0.007	−0.013	*G*1·*S*15 + (G2·*S*8 + G3·*S*12 + G4·*S*8^2^ + G5·*S*12^2^ + G6·*S*8·*S*12)
MCP4-5_A	0.214	−0.091	0.004	0.250	0.005	−0.011	*G*1·*S*19 + (G2·*S*12 + G3·*S*16 + G4·*S*12^2^ + G5·*S*16^2^ + G6·*S*12·*S*16)

Two calibration trials are used for CMC1 flexion/extension (CMC1_F), leading to a linear relationship with sensor 1 (Fig. 1, right) and to an adjustment factor related to the sensor 4 movement. Similarly, two calibration trials are used for CMC1 abduction (CMC1_A), considering a linear relationship with sensor 4 and an adjustment factor related to sensor 1 movement. Finally, two postures are needed for a palmar arch (CMC5_F), which is estimated from sensor 20 assuming a linear relationship. The sensor gains for the thumb CMC and palmar arch are shown in Table 5.

**Table 5:** Information on the sensor gains used for the calibration of the thumb CMC and palmar arch gauges. Sx corresponds to the sensor number, according to Fig. 1.

	Sensor gains		Anatomical angle calculation
	G1	G2	
CMC1_F	0.713	0.623	G1*S1 + G2*S4
CMC1_A	−0.321	0.050	G1*S4 + G2*S1
CMC5_F	0.285	—	G1*S20

**Fig. 4:**
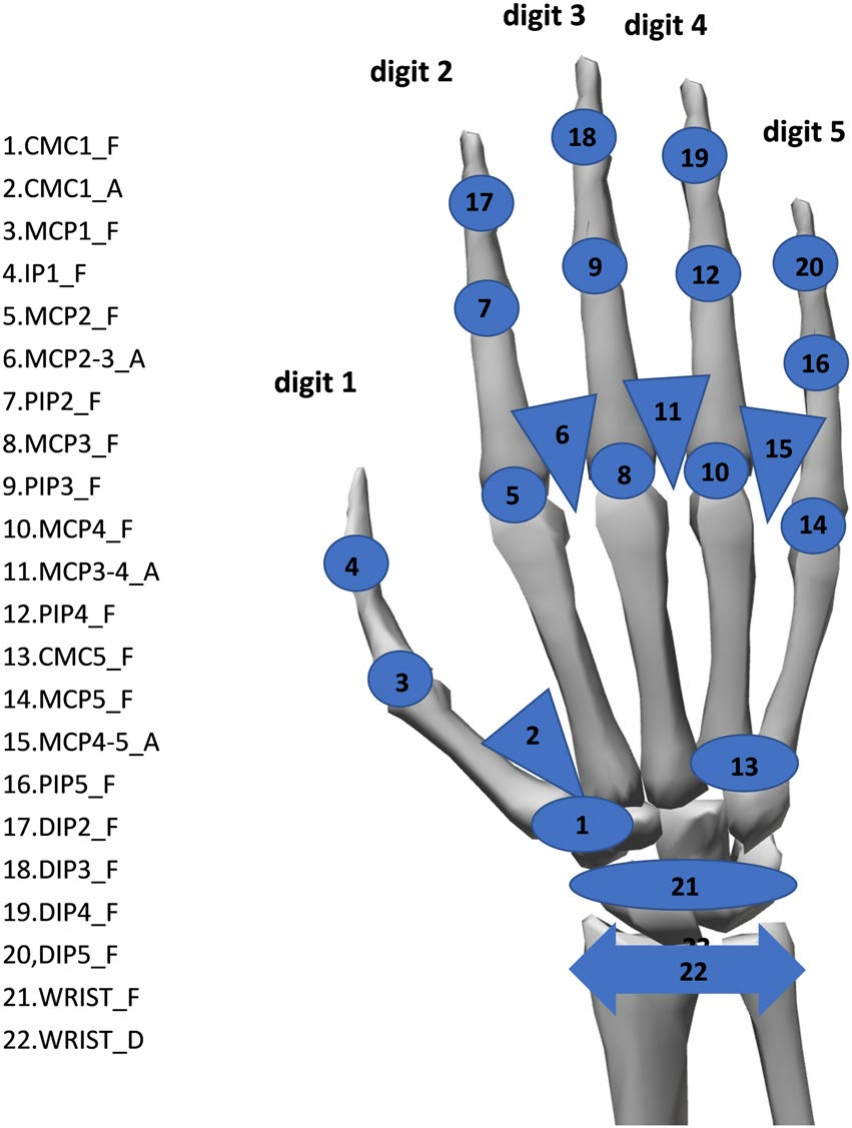
List of recorded anatomical angles. Nomenclature: _D for deviation (double arrow), _F for flexion/extension (circles or ellipses in the image), _A for abduction/adduction (triangles in the image); 1 to 5, digits.

**Fig. 5:**
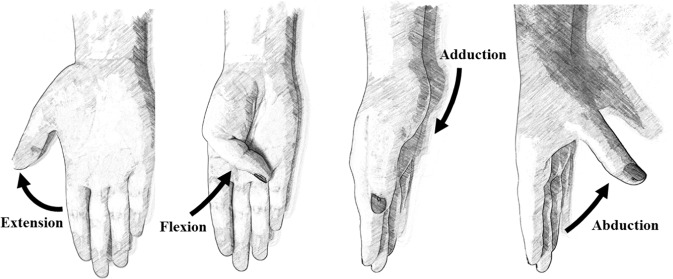
Movements of the carpometacarpal joint.

The calibration protocol was recorded on 10 subjects. Afterwards, the gains obtained for each sensor were used to compute the joint angles for all the subjects. In order to do this, the sensor signals were transformed according to the outputs relative to a reference posture. The reference posture was chosen as the central part of movement 11 in exercise B (Fig. 2), corresponding to the hand resting on the table with the fingers closed together and extended. The anatomical angles resulting from the calibration phase are represented in Fig. 4. The defined sign criteria are shown in Table 6 and Fig. 5.

**Table 6:** Sign criteria considered.

DIP(2-5)_F, PIP(2-5)_F, IP1_F, MCP(1-5)_F	Flexion+/Extension−
WRIST_F	Flexion+/Extension−
WRIST_D	Radial deviation+/Cubital deviation−
MCP(2-3, 3-4, 4-5)_A	Fingers separated+/Fingers together−
P_Arch (CMC5_F)	Flexion+/Extension−
CMC1_F	Flexion+/Extension− (See Fig. 5)
CMC1_A	Abduction+/Adduction− (See Fig. 5)

## Data Records

The kinematic data produced with the described methods are stored on Zenodo^19^ (a general-purpose open-access repository developed under the European OpenAIRE program and operated by CERN) and on Ninapro (a repository with publicly available data resources to improve robotic hands and prostheses control, http://ninapro.hevs.ch/) as Ninapro DB9 dataset. The format and content for data sets are described hereafter. For each subject and exercise, the database contains one.mat file (http://www.mathworks.com/) with synchronized variables. The variables included in the files are:

- subject: subject number;
- exercise: exercise number;
- glove (22 columns): uncalibrated signal from the 22 sensors of the Cyberglove. Details on the location of the sensors are described in Fig. 1;
- angles (22 columns): calibrated signal from the 22 sensors of the Cyberglove by applying the post-processing calibration procedure^17^.
- re-stimulus (1 column): the a-posteriori refined label of the movement;
- re-repetition (1 column): re-stimulus repetition index;
- stimulus (1 column): the label of the movement;
- repetition (1 column): stimulus repetition index;
- order of angles (22 columns): name of the angles corresponding to variable “angles”.

## Technical Validation

In order to allow better modelling of hand kinematics (to improve robotics, 3D modelling, rehabilitation, medicine and neuroscience) the data needs to correspond to data acquired in real life conditions. They need to correspond as much as possible to the movements included in the acquisition protocol and they should not be affected by experimental conditions.

The technical validation is thus performed in two phases. The first phase aims at verifying that the calibrated data correspond to the movements included in the acquisition protocol. The second phase verifies that the data are similar to data produced in real life by evaluating the effect of experimental conditions (such as movement repetition, movement number and subject number) on the range of the joint angles.

### Correspondence to the recorded data

In order to verify that the calibrated data correspond to the movements included in the acquisition protocol, we computed a graphic representation of each average posture. For each movement and for all the subjects, all movement repetitions (as identified after the re-labelling) were divided into three parts. While the first part of the movement in general corresponds to reaching the posture targeted within the movement and the last part of the movements corresponds to getting back to the rest position, the central parts are the ones that are statistically expected to correspond the most to the target posture. The average postures were thus computed as the mean value of the central (second) part among all the subjects. Figures 2 and 3 present the average postures as obtained with the described procedure using a publicly available OpenSim model of the hand skeleton (10.13140/RG.2.2.13115.21282). The visual inspection of the average graphic representations allows us to verify that the obtained data correspond and closely resemble the postures expected according to the acquisition protocol. When the fist is closed (exercise B, movement 6) there is no full correspondence between the original movement and the computed average model. The central part of the movement (considered to compute the visual representation) may in a few cases involve parts of the reaching phase, as well as of the phase in which the hand gets back to the rest position.

### Effect of experimental conditions on the hand kinematics

**Fig. 6:**
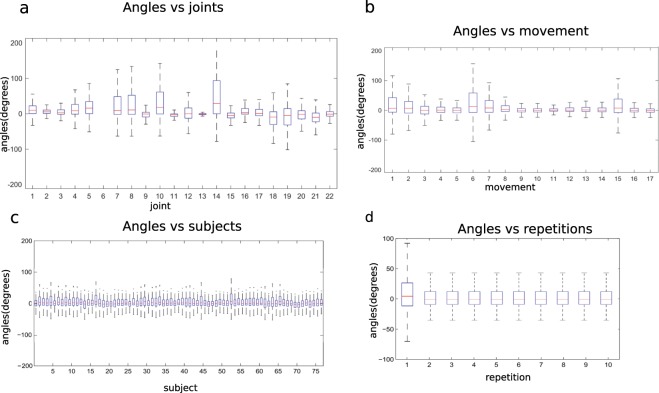
Effect of experimental conditions on the hand kinematics for the Exercises B. Subplots represent different experimental conditions: joints (subplot **a**) referred as 1 to 22, according to Fig. 4; movement (subplot **b**); subject (subplot **c**); movement repetition (subplot **d**). The horizontal central mark in the boxes is the median; the edges of the boxes are the 25^th^ and 75^th^ percentiles; the whiskers extend to 1.5 times the interquartile range.

**Fig. 7:**
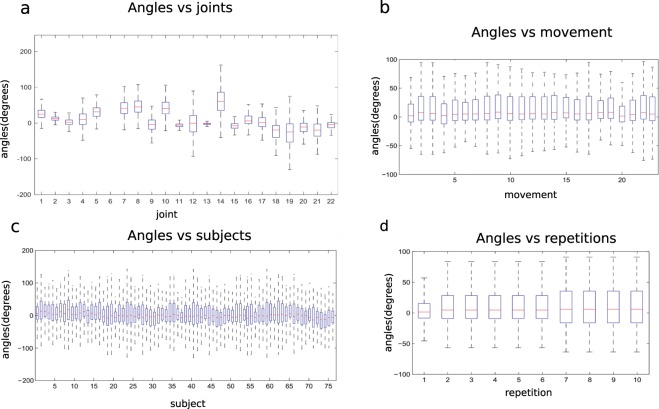
Effect of experimental conditions on the hand kinematics for the Exercises C. Graphical representation of each movement is shown in subplot a. Different subplots represent different experimental conditions: joints (subplot **a**) referred as 1 to 22, according to Fig. 4; movement (subplot **b**); movement repetition (subplot **c**); subject (subplot **d**). The horizontal central mark in the boxes is the median; the edges of the boxes are the 25th and 75th percentiles; the whiskers extend to 1.5 times the interquartile range.

In order to verify that the data are similar to data produced in real life, the effect of experimental factors on the range of the joint angles was evaluated. Factors that can affect the joint angles are the joint (since each one corresponds to a specific sensor), the movement, the subject or the movement repetition. This analysis was performed separately for the exercises included in the dataset and are reported in Figs. 6 and 7.

In exercise B, a large variability can be noticed when considering how the angles change in relation to the joints. In particular, the MCPs present a positive median with the largest range of motion, together with the PIP of the index finger. Considering angles vs. movements, the movements 1, 6, 7 and 15 present the widest range of motion, which is in agreement with the fact that these movements include flexion of at least 4 fingers, since they include at least partially a fist. Considering angles vs. subjects, variability is limited among subjects. Considering angles vs. movement repetitions, it is interesting to notice that the first movement repetitions present a wider range of motion, probably because this is the first-time subjects are performing the task and the movements are not linked to specific objects that can limit the movement of the hand across different repetitions.

For the exercise C, visualizing the angles for each joint, it is observed that the MCPs present a positive median with a large range of motion, together with the PIP of the fingers.

In general, all movements present a range of motion that is wider than in exercise B. This is logical since these are grasps and functional activities that need more complex joint movements. Considering angles vs. subjects, variability is limited among subjects also for this exercise. Differently from exercise B, the first repetition presents a smaller range of motion. This may be due to the higher complexity of the tasks, which can require more attention and results in slower motion by the subjects. In this exercise, the movements are linked to specific objects that limit the movement of the hand across repetitions.

In conclusion, it is clear from these results that the Ninapro DB9 kinematic dataset appears as a reliable resource to improve current scientific advancement in robotics, rehabilitation, prosthetics, etc.

## Usage Notes

Many factors can affect the amplitude of the signal from the sensors, including the acquisition setup, the anatomical characteristics of the subject and fatigue.

Sensor 11 (that corresponds to MCP2-3_A) was not included in the calibrated data due to noise problems.

DIP sensors provide reliable angles when a subject’s hand size is large (i.e. when the glove properly fits the hand). They may provide partial results when the hand of the subject is small. Therefore, attention needs to be taken when using the information.

Sensor gains specified in this manuscript are only valid for the Cyberglove used during these experiments are not valid for any other glove.

The number of subjects included in the dataset (77) is not high by a statistical point of view, but it is high in comparison to the number of subjects included in previous studies on kinematic data (which usually include up to 10 subjects). To our knowledge, this dataset is currently the biggest publicly available dataset of kinematic data. Regarding gender factors, the male/female ratio of the collected data is 2.7 with 21 females of the 77 subjects. A higher number of female participants can provide additional value for studies in which correlation between sex and hand movements are studied. However, we cannot currently record additional subjects. So, for such studies we suggest users to create matched samples choosing subjects from the dataset.

## Data Availability

The Matlab code used to calculate hand joint angles from CyberGlove instrumented gloves raw data can be accessed as open access^29^.
